# Restoration and maintenance of segment lordosis in oblique lumbar interbody fusion

**DOI:** 10.1186/s12891-022-05855-z

**Published:** 2022-10-14

**Authors:** Ke Gong, Yang Lin, Zhibin Wang, Feng Li, Wei Xiong

**Affiliations:** Department of Orthopaedics, Tongji Hospital affiliated to Tongji Medical College of Huazhong, University of Science & Technology, No.1095 Jie Fang Avenue, Wuhan, China

**Keywords:** Oblique lumbar interbody fusion, Segment lordosis angle, Cage subsidence, Lumbar degenerative disease

## Abstract

**Purpose:**

Restoration of the segment lordosis angle (SLA) can effectively reduce the risk of adjacent segment degeneration. This study aimed to perform a comprehensive multifactor analysis of the risk factors affecting restoration and maintenance of the SLA in oblique lumbar interbody fusion (OLIF).

**Methods:**

Seventy-three patients (93 segments) who underwent OLIF with posterior pedicle screw fixation due to lumbar degenerative disease between January 2015 and December 2019 were included. Radiographic parameters including the middle disc height (MDH), segment lordosis angle (SLA), cage center point ratio (CPR), cage subsidence, and L1 CT Hounsfield Unit (HU) were measured.

**Results:**

The postoperative SLA increased from 3.5° to 8.7°, and decreased to 6.7° at the last follow-up. Multivariate analysis showed that preoperative SLA, CPR and cage subsidence were significantly correlated with SLA restoration. The significant correlations were between restoration of SLA with pre-operative SLA (r=-0.575, adjusted R2 = 0.323, *P* < 0.01) and between SLA restoration and CPR (r = 0.526, adjusted R2 = 0.268, *P* < 0.01). Cage subsidence was found in 12.9% (12/93) of segments and was the main factor affecting SLA loss (4.2 ± 1.0° versus 1.7 ± 2.1°, P < 0.01). Logistic regression analysis showed that CPR < 50%, L1 CT HU < 110 and cage height > preoperative MDH were risk factors for cage subsidence. Cages placed anteriorly (CPR ≥ 50%) showed a large SLA increase and lower incidence of cage subsidence than those placed posteriorly (5.9 ± 3.9° versus 4.2 ± 3.2°, P < 0.05; 1.8% versus 28.9%, P < 0.05, respectively).

**Conclusion:**

SLA restoration is dependent on preoperative SLA, cage subsidence and cage position in OLIF. Cage position is the key determinant of SLA restoration and placement of the cage at the anterior position (CPR ≥ 50%) can achieve better restoration of the SLA and reduce the incidence of cage subsidence.

## Introduction

Restoration of the segment lordosis angle (SLA) is important in lumbar interbody fusion (LIF) because adequate restoration of the SLA can effectively reduce the risk of adjacent segment degeneration (ASD) [1–4]. However, restoration of the SLA through the commonly used of posterior/transforaminal lumbar interbody fusion (P/TLIF) procedure has been disappointing especially in the lower lumbar segment, in which bilateral facet resection or a pair of cages is required for further restoration of the SLA [5].

Anterior/lateral lumbar interbody fusion (A/LLIF) may provide better restoration of disk height (DH) and SLA with insertion of a large interbody cage [6, 7]. However, the risk of nerve and vascular injury limits the use of A/LLIF [8]. Oblique lumbar interbody fusion (OLIF) was first reported in 2012 and has been widely used for the treatment of lumbar degenerative diseases in recent years. It is a minimally-invasive lateral anterior approach through the physiological gap between the aorta and psoas major and previous studies have reported that it can achieve satisfactory outcomes with the advantages of less surgical trauma, quick recovery and indirect decompression [9–13].

However, the hypothetical advantage is that insertion of large interbody cage in OLIF does not result in better SLA restoration than that obtained with T/PLIF [12, 14]. It is particularly important to investigate the risk factors affecting SLA restoration in OLIF. A previous study reported that variously risk factors such as cage position, cage size (height, width, angle) and cage subsidence were associated with restoration or maintenance of the SLA in T/PLIF [15–18]. However, most studies have evaluated one of the variables and consequently, this study aimed to conduct a comprehensive multifactor analysis of the risk factors affecting restoration and maintenance of the SLA in OLIF.

## Materials and methods

### Study population

From January 2015 to December 2019, consecutive patients who underwent OLIF surgery at the Spine Center of Tongji Hospital due to lumbar spondylolisthesis, lumbar spinal stenosis, disc herniation, degenerative scoliosis, or other lumbar degenerative diseases were enrolled. The exclusion criteria were as follows: (1) duration of follow-up < 12 months; (2) presence of other lumbar diseases, such as tumor, infection, or fracture; (3) patients without posterior pedicle screw fixation; and (4) patients who underwent osteotomy or facetectomy.

### Surgical technique

All patients underwent OLIF in the right lateral decubitus position. A 4–5 cm oblique incision 1/3 below and 2/3 above the anterior projection of the disc onto the skin was made. The external oblique, internal oblique, and transverse abdominal muscles were dissected bluntly. Using fingers and handheld retractor to retract the retroperitoneal fat, ureter and abdominal content. Then gently retracted the psoas muscle. Cages (Clydesdale PEEK; Medtronic, Memphis, TN, USA) were inserted together with an allogeneic bone graft after discectomy and endplate preparation. Posterior fixation was performed by using percutaneous pedicle screws.

### Clinical and radiographic assessments

Clinical efficacy was assessed using the visual analog scale (VAS) score, the Japanese Orthopedics Association (JOA) score, and the Oswestry Disability Index (ODI) preoperatively and at the last follow-up.

Radiographs were taken preoperatively, postoperatively (3 day after the operation) and at the last follow-up. All images were collected from the Synapse system and Surgimap software (version 2.3) was used for the measurement of sagittal parameters. Two experienced spine surgeons were trained to improve the measurement consistency and all the parameters were measured twice individually. To achieve ideal measurements, we evaluated the parameters on radiographs magnified 150% [19], and the discussion will be performed to achieve consensual when there is disagreement regarding intraoperative endplate injury or cage subsidence.

**Fig. 1:**
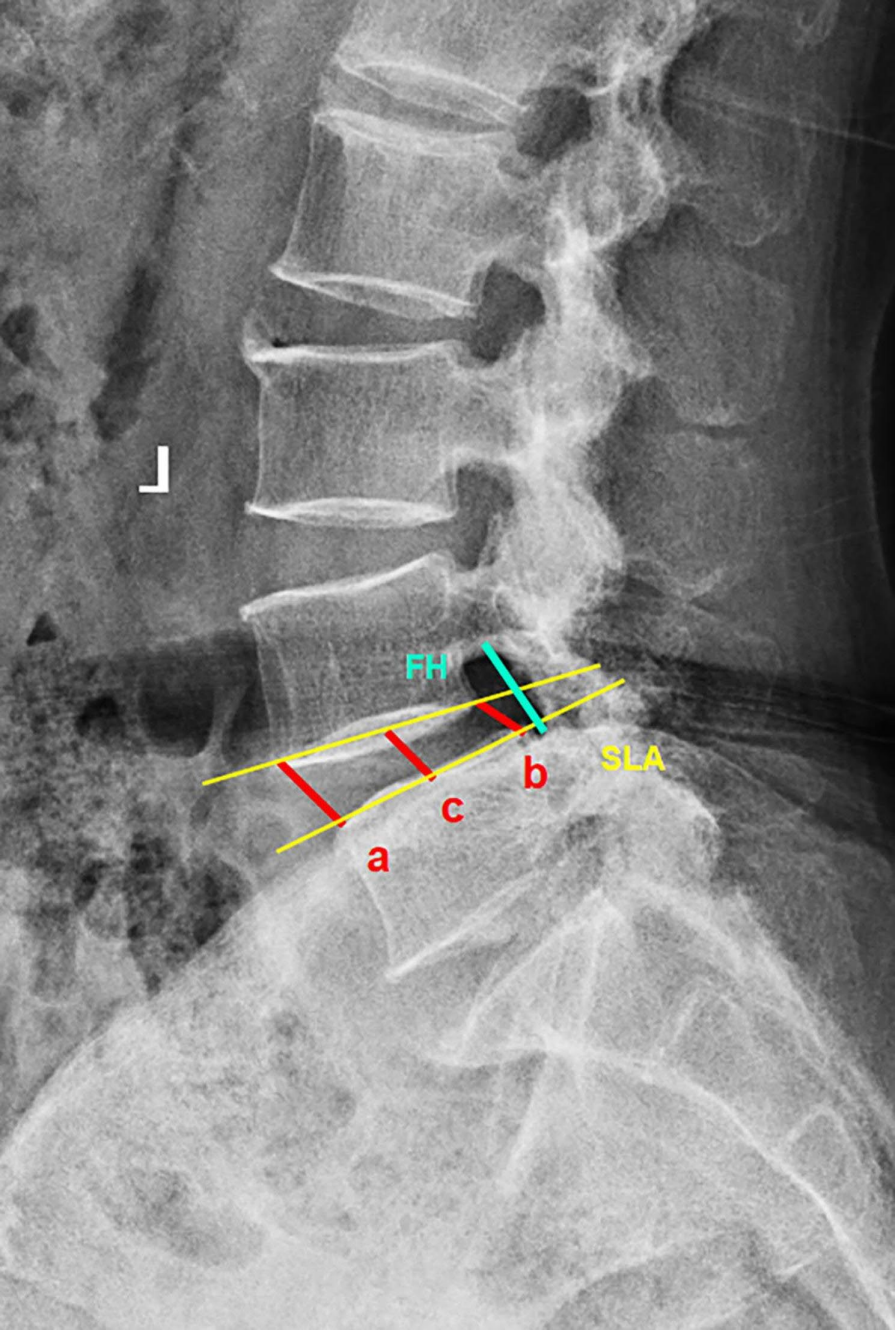
Measurements of radiographic parameters. disc height (a: ADH, anterior disc height, b: PDH, posterior disc height, c: MDH, middle disc height), segment lordosis angle (SLA), and foraminal height (FH).

**Fig. 2:**
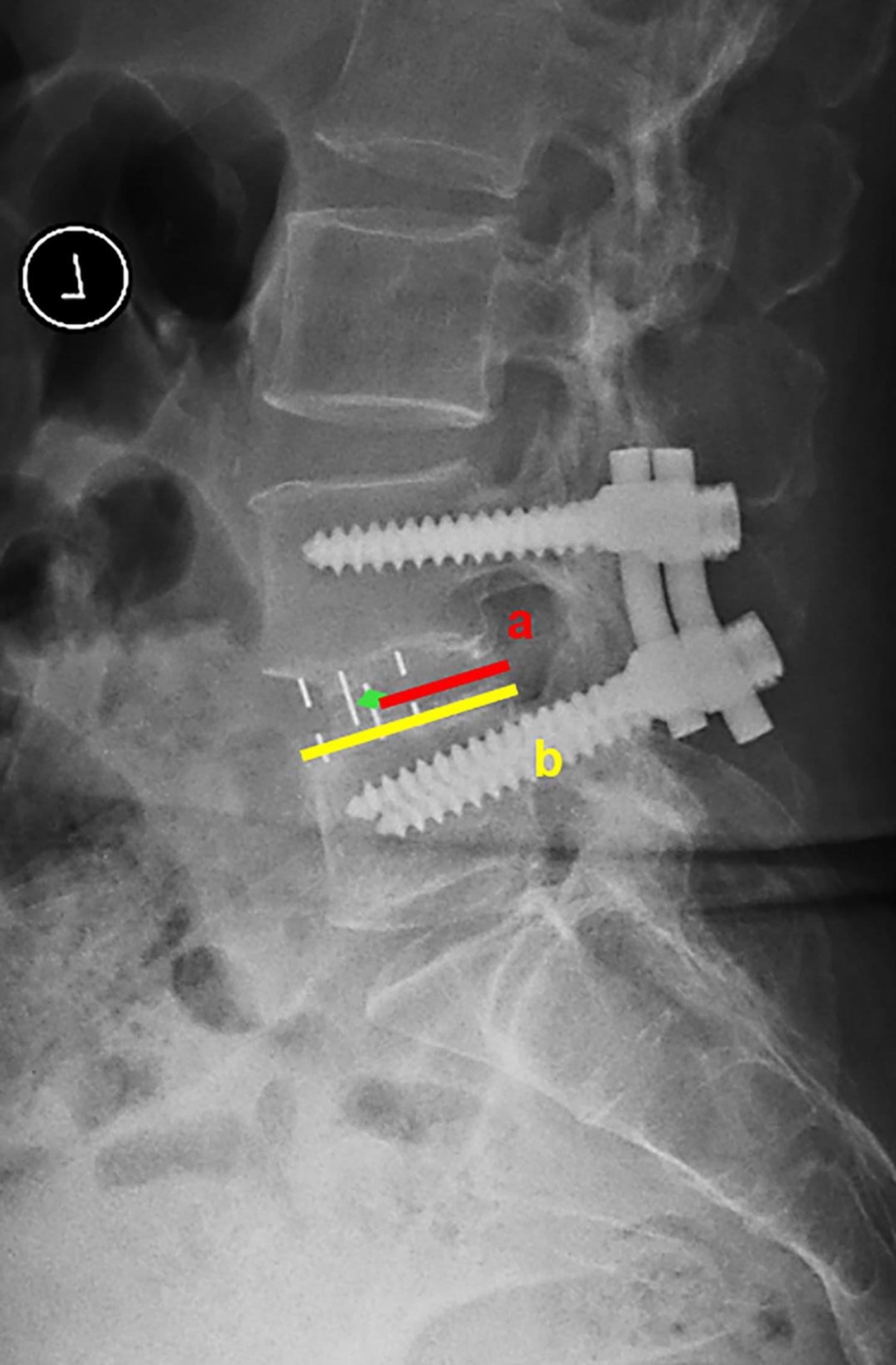
The measurements of the cage central point ratio (CPR). CPR = 100%×a/b.

Disc height (DH), foraminal height (FH) and SLA were measured on lumbar lateral radiographs at preoperatively, postoperatively, and at the last follow-up, as shown in Fig. 1. DH (A/M/PDH) was defined as the distance from the upper endplate to the lower endplate at the anterior/middle/posterior position. FH was defined as the pedicle distance between surgical segments. SLA was defined as the angle between the lower endplate and the upper endplate at the surgical segment. Intraoperative endplate injury was defined as discontinuity of the endplate contour affected by the cage on postoperative lumbar lateral X-ray [20]. Cage subsidence was defined as cage migration of more than 2-mm into the adjacent vertebral body at the last follow-up [21]. The cage center point ratio (CPR) was defined as the ratio between the distance from the center of the cage to the back of the intervertebral space and the length of the upper endplate of the lower vertebral body [15], as shown in Fig. 2. Bone mineral density (BMD) was assessed through preoperative L1 CT Hounsfield Unit (HU) [18].

### Statistical analysis

All statistical tests were performed using SPSS, version 21 (IBM Corporation., Armonk, NY, USA). Continuous variables are shown as the mean ± standard deviation. The normality test was performed using the Kolmogorov–Smirnov test. Multifactor regression analysis was performed to investigate the influencing factors of SLA restoration. The correlation between restoration of the SLA and the preoperative SLA and CPR was evaluated using the Pearson correlation coefficient. To identify the risk factors for cage subsidence, univariable logistic regression analysis was used to assess the correlation of variables and cage subsidence (or no- cage subsidence). For the statistically significant factors, multivariate logistic regression analysis was used to further calculate the odds ratios (ORs). The independent sample t test and chi-square test or Fisher’s exact test were used to compare the difference between the anterior and posterior groups. P values of < 0.05 were considered statistically significant.

## Results

### Demographics and clinical results

A total of 73 patients with 93 OLIF segments were included in this study. The mean age of the patients was 56.2 ± 12.3 years with a mean 20.8 ± 10.6 months follow-up. The demographic and surgery-related data of the 73 patients are shown in Table 1. Satisfactory clinical outcomes were obtained at the last follow-up. The VAS scores for back pain and leg pain decreased from 5.8 ± 1.4 and 5.1 ± 1.9 (preoperative) to 1.5 ± 0.9 and 0.5 ± 0.6 (last follow-up), respectively. The ODI decreased from 54.6 ± 11.2 to 12.3 ± 6.0, and the JOA score increased from 13.7 ± 3.2 to 25.2 ± 2.0.

**Table 1:** Demographic and surgical data of the 73 patients

**Demographic data**		
Age (years), mean ± SD	56.2 ± 12.3	
Sex: male/female (n)	20/53	
Follow-up (months), mean ± SD	20.8 ± 10.6	
Pre-operation L1 CT HU	124.2 ± 42.8	
Primary Diagnosis, (%)	n = 73	
Lumbar spondylolisthesis	34 (46.6%)	
Lumbar stenosis	15 (20.5%)	
Degenerative scoliosis	4 (5.5%)	
Lumbar disc herniation	18 (24.7%)	
Adjacent segment disease	2 (2.7%)	
**Surgical data**		
OLIF level	n = 93	
L2-3	5 (5.4%)	
L3-4	20 (21.5%)	
L4-5	67 (72.0%)	
L5-S1	1 (1.1%)	
Cage size	n = 93	
10 × 22 mm 6°	17 (18.3%)	
12 × 22 mm 6°	76 (81.7%)	

*73 OLIF patients with 93 segments

### Radiographic results

The A/M/PDH, FH, and SLA were significantly increased from 13.0 ± 3.5, 11.2 ± 2.4, 10.1 ± 2.4, 21.5 ± 2.8 mm, 3.5 ± 4.8° (preoperative) to 17.6 ± 2.6, 14.2 ± 2.0, 11.6 ± 2.0, 25.5 ± 2.6 mm, 8.7 ± 3.4°(post-operative), respectively(*P* < 0.05). However, those of parameters were significantly decreased at last follow-up (15.7 ± 3.4, 12.9 ± 2.2, 10.7 ± 1.8, 23.7 ± 2.5 mm, 6.7 ± 4.3°, respectively, *p* < 0.05) compared with those at the postoperative timepoint.

**Fig. 3:**
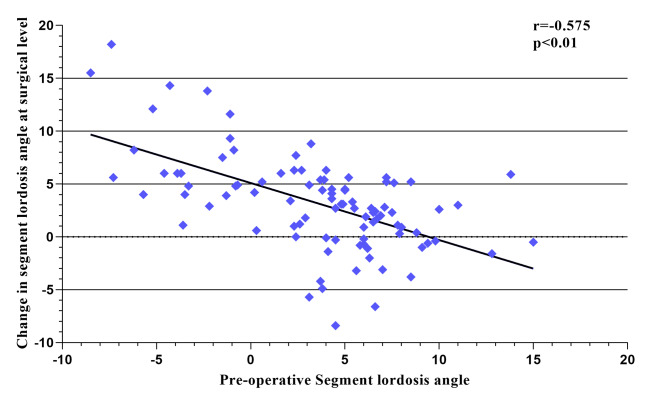
Relationship between the change in the segment lordosis angle (SLA) and the preoperative SLA (r=-0.575, adjusted R2 = 0.323, *P* < 0.01)

**Fig. 4:**
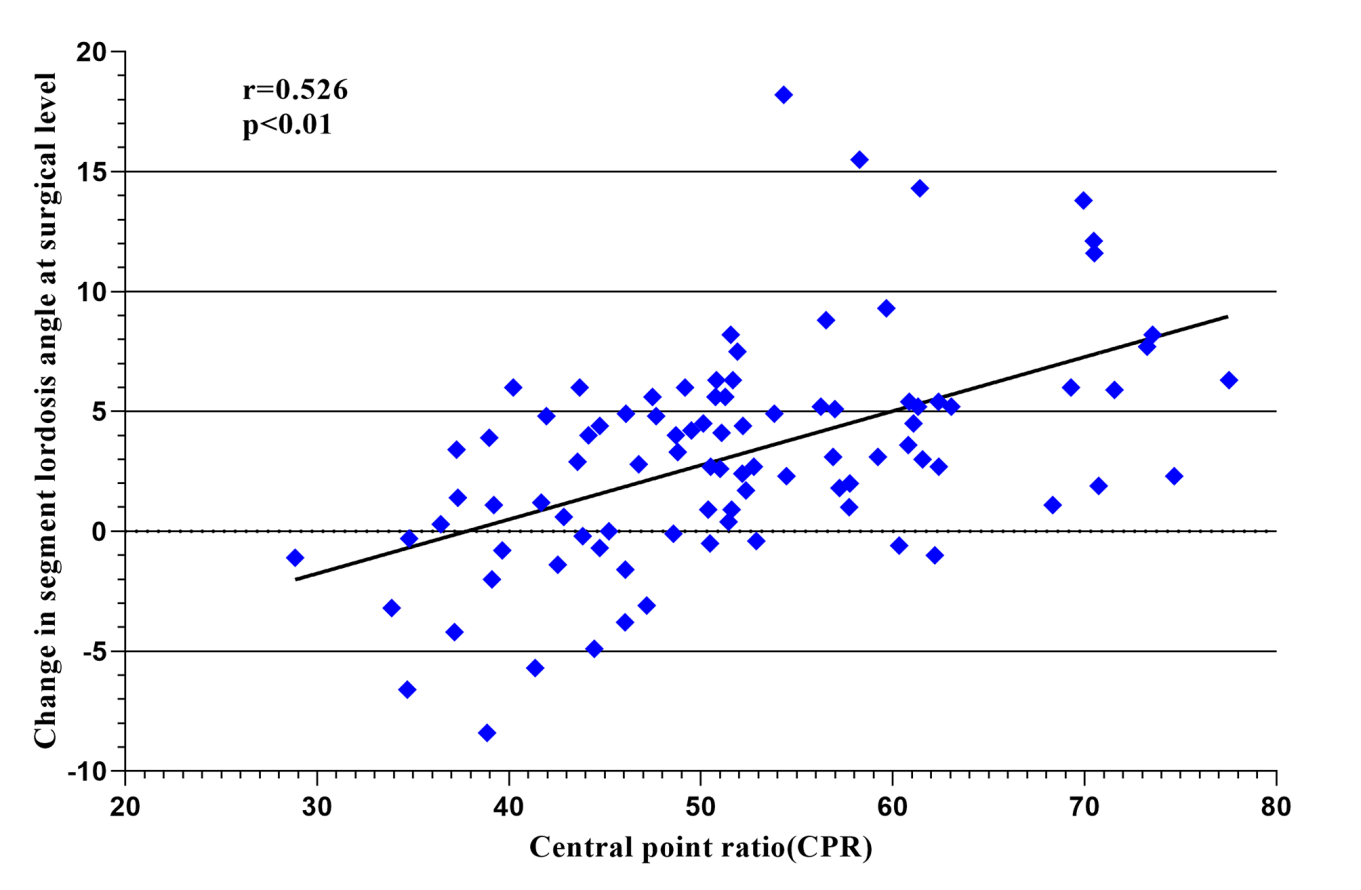
Relationship between the change in the segment lordosis angle (SLA) and the cage central point ratio (r = 0.526, adjusted R2 = 0.268, *P* < 0.01)

Multivariate regression analysis showed that preoperative SLA, CPR and cage subsidence were significantly correlated with restoration of the SLA at last follow-up (Table 2). Intraoperative restoration of the SLA was significantly correlated with the preoperative SLA (r=-0.575, adjusted R2 = 0.323, *P* < 0.01, Fig. 3) and CPR (R = 0.526, adjusted R2 = 0.268, *P* < 0.01, Fig. 4). Cage subsidence was found in 12 (12.9%) segments and the SLA loss in the subsidence segment was significantly higher than that in the no-cage subsidence segment (4.1 ± 1.3° versus 1.7 ± 2.1°, *P* < 0.01). Logistic regression analysis was conducted to identify risk factors for cage subsidence and CPR < 50%, cage height > preoperative MDH, L1 CT HU < 110 were significantly associated with cage subsidence, as shown in Table 3.

**Table 2:** Multiple line regression analysis of predictors of postoperative segment lordosis angle restoration

	B	SE	Beta	t Value	P Value
**Age**	0.021	0.031	0.058	0.696	0.489
**Gender**	0.899	0.717	0.088	1.254	0.213
**Spondylolysis**	0.841	0.652	0.084	1.289	0.201
**L1 CT HU**	0.005	0.009	0.047	0.560	0.577
**Cage height**	-0.279	0.815	-0.025	0.360	0.719
**Preop-SLA**	-0.628	0.070	-0.678	-8.932	0.000*
**CPR**	19.659	2.896	0.464	6.787	0.000*
**Cage subsidence**	-3.067	1.018	-0.231	-3.014	0.003*

CT HU, CT Hounsfield unit; SLA, segment lordosis angle; CPR, cage central point ratio. *, *P* < 0.05

**Table 3:** Logistic regression analysis of risk factors associated with Cage subsidence

	Univariable		Multivariable	
Variables	OR1(95%CI)	P value	OR2(95%CI)	P value
Age				
< 60 years old	Reference			
≥ 60 years old	0.9(0.3-3.0)	0.842		
Gender				
Male	Reference			
Female	0.5(0.1–2.6)	0.444		
Number of OLIF level				
< 3	Reference		Reference	
3	4.6(1.1–18.6)	0.034*	0.6(0.1–3.8)	0.557
L1 CT HU				
≥ 110	Reference		Reference	
< 110	9.5(1.9–46.2)	0.005*	9.0(1.3–64.2)	0.028*
Cage height				
10 mm	Reference			
12 mm	2.7(0.3–22.5)	0.357		
The difference of CH to preoperative MDH				
CH ≤ preoperative MDH	Reference			
CH > preoperative MDH	10.2(1.3–82.8)	0.030*	11.6(1.2-114.3)	0.035*
CPR				
CPR ≥ 50%	Reference			
CPR < 50%	22.0(2.7-178.4)	0.004*	24.7(2.5-241.2)	0.006*

OR, Odds ratio; CT HU, CT Hounsfield unit; MDH, middle disc height; CPR, cage central point ratio. * *P* < 0.05, difference was significant

**Fig. 5:**
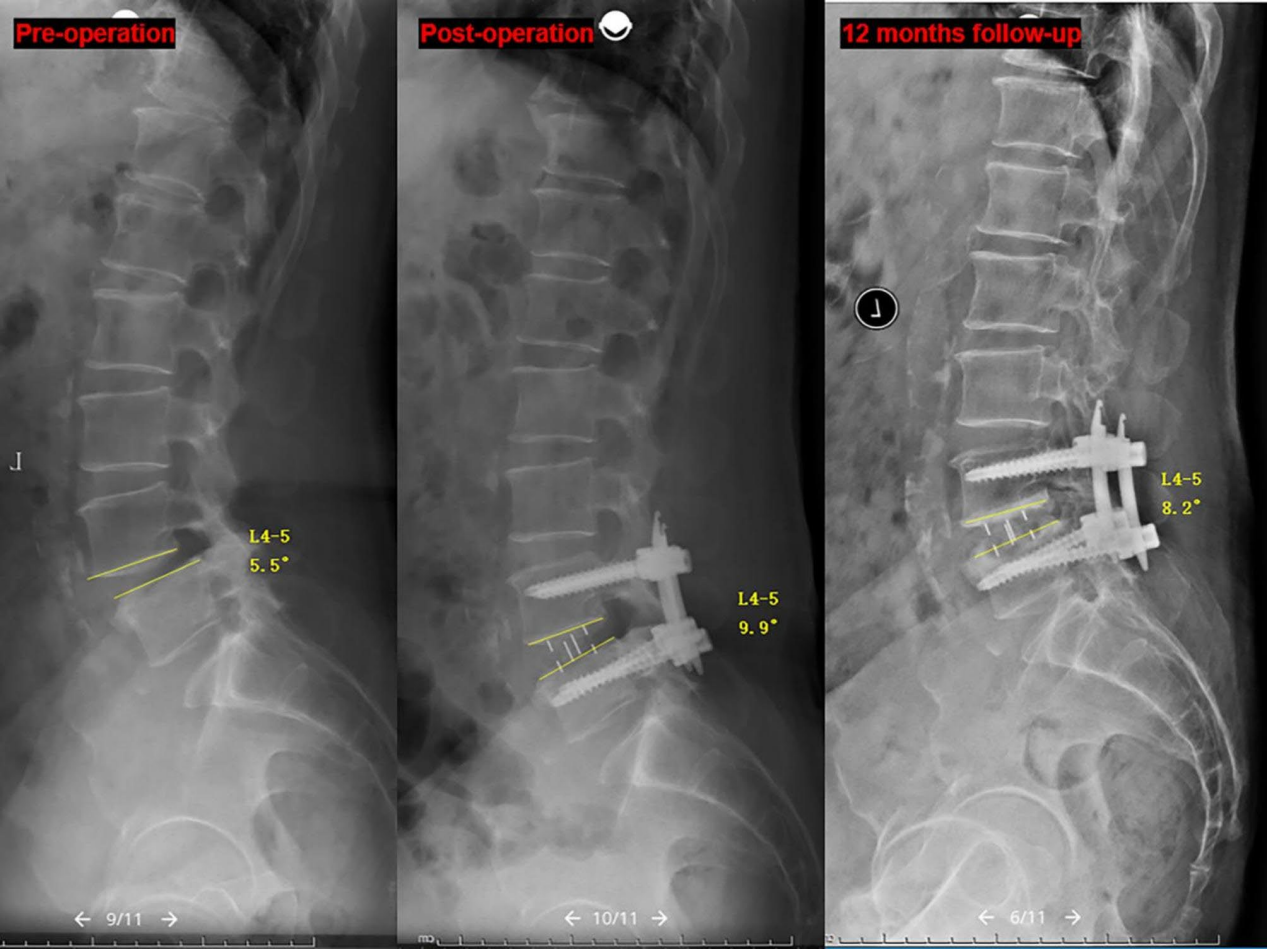
A 67 year-old male underwent L4-5 OLIF due to degenerative spondylolisthesis, CPR = 53%. The SLA was increased from 5.5° to 9.9° and intraoperative endplate injury was observed postoperatively. The SLA decreased to 8.2° at the 12-month follow-up

**Fig. 6:**
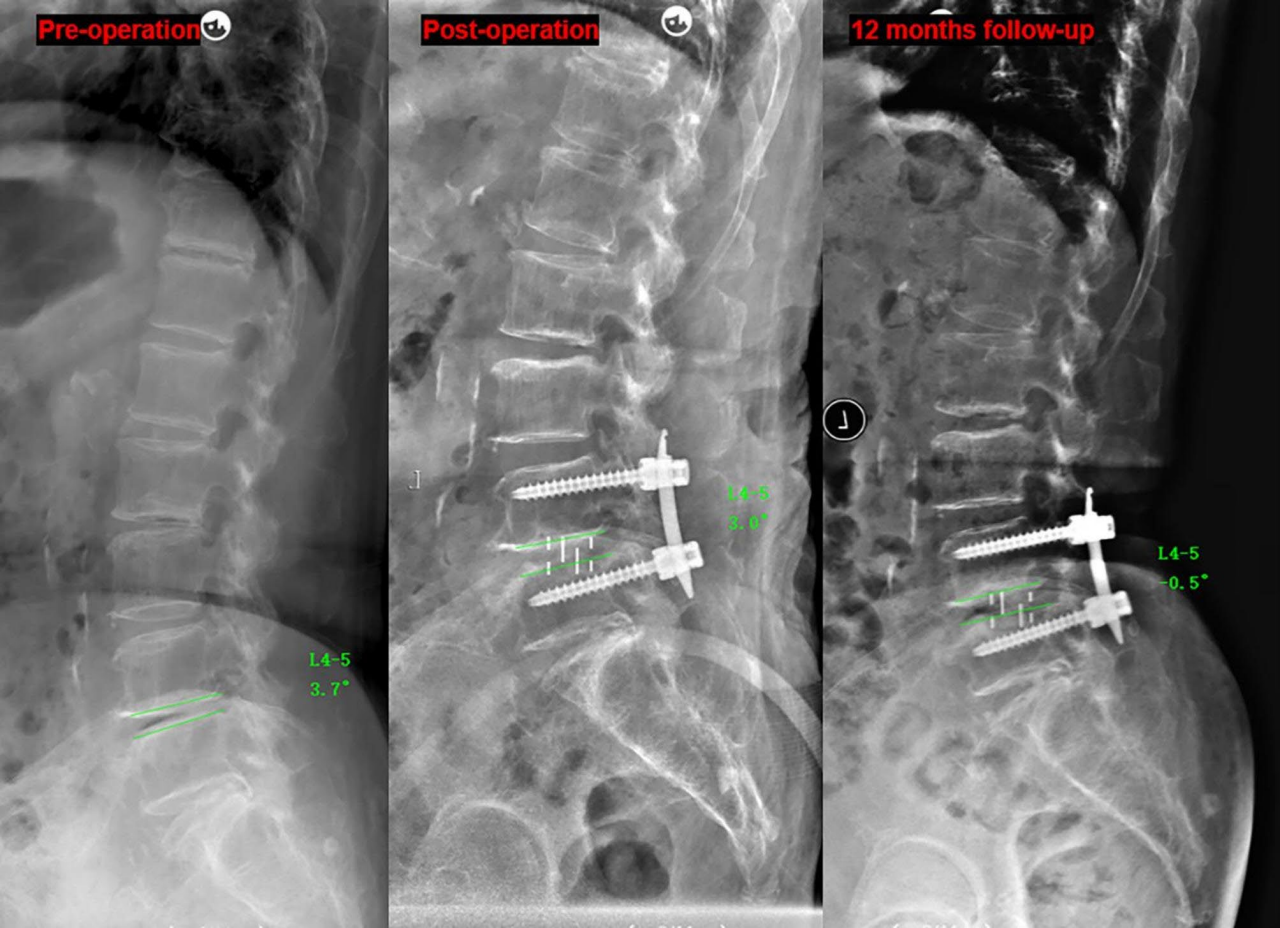
A 75 year-old female underwent L4-5 OLIF due to lumbar spinal stenosis, CPR = 37%. The SLA was changed from 3.7° to 3.0° and the intraoperative endplate injury was observed postoperatively. The SLA decreased to -0.5° at 12-month follow-up

The CPR was significantly correlated with SLA restoration and cage subsidence. Therefore, the segments were divided into two groups according to the CPR (anterior group: CPR ≥ 50%, 55 segments; posterior group: CPR < 50%, 38 segments). There were no significant differences between the two groups with respect to age, sex, cage size, follow-up time and preoperative SLA, A/M/PDH, FH, or L1 CT HU (*P* > 0.05). The radiographic parameters according to time are shown in Table 4. The intraoperative increase in the SLA was significantly large (5.9 ± 3.9° versus 4.2 ± 3.2°, *P* = 0.021) while the intraoperative increased of PDH and FH were significantly smaller in the anterior group (0.8 ± 2.3 mm versus 2.6 ± 2.4 mm, *P* = 0.000; 3.6 ± 2.2 mm versus 4.7 ± 2.2 mm, *P* = 0.014, respectively). The incidence of intraoperative endplate violation and cage subsidence in the posterior group was significantly higher (18.4% versus 3.6%, *P* < 0.05; 28.9% versus 1.8%, *P* < 0.05, respectively) and it caused significantly higher loss of A/MDH, FH and SLA compared with the anterior group(*P* < 0.05). The loss of SLA in the intraoperative endplate violation segment was significantly higher in the posterior group than in the anterior group (4.3 ± 1.1° versus 1.0 ± 1.0°, *P* < 0.05), and two cases are shown in Figs. 5 and 6.

**Table 4:** Comparison of radiographic parameters between anterior and posterior group at Pre-, Post- after operation and final follow-up

	Anterior group (CPR ≥ 50%, 55 level)	Posterior group (CPR < 50%, 38 level)	P value
ADH (mm)			
Base line	13.4 ± 3.7	12.3 ± 3.1	0.154
Post-operation	18.1 ± 2.7	17.0 ± 2.4	0.041*
Follow-up	17.1 ± 3.1	13.7 ± 2.7	0.000*
Post-operation increased	4.7 ± 3.0	4.6 ± 2.2	0.885
Follow-up decreased	1.0 ± 2.0	3.3 ± 2.3	0.000*
MDH (mm)			
Base line	11.4 ± 2.3	10.9 ± 2.4	0.331
Post-operation	14.2 ± 1.8	14.3 ± 2.3	0.707
Follow-up	13.3 ± 2.1	12.3 ± 2.1	0.027*
Post-operation increased	2.8 ± 2.0	3.4 ± 2.3	0.157
Follow-up decreased	0.8 ± 1.7	2.0 ± 1.9	0.002*
PDH (mm)			
Base line	10.3 ± 2.3	9.8 ± 2.5	0.291
Post-operation	11.1 ± 2.0	12.3 ± 2.1	0.003*
Follow-up	10.4 ± 1.7	11.1 ± 2.0	0.080
Post-operation increased	0.8 ± 2.3	2.6 ± 2.4	0.000
Follow-up decreased	0.7 ± 1.4	1.3 ± 2.0	0.107
FH (mm)			
Base line	21.3 ± 2.9	21.8 ± 2.8	0.407
Post-operation	24.9 ± 2.4	26.5 ± 2.6	0.002*
Follow-up	23.3 ± 2.5	24.3 ± 2.7	0.066
Post-operation increased	3.6 ± 2.2	4.7 ± 2.2	0.014*
Follow-up decreased	1.6 ± 1.4	2.2 ± 1.6	0.044*
SLA(Degree)			
Base line	4.1 ± 4.9	2.6 ± 4.6	0.157
Post-operation	10.0 ± 3.0	6.8 ± 3.0	0.000*
Follow-up	9.1 ± 2.9	3.2 ± 3.7	0.000*
Post-operation increased	5.9 ± 3.9	4.2 ± 3.2	0.026*
Follow-up decreased	0.9 ± 1.2	3.5 ± 2.3°	0.000*
Intraoperative endplate injury	3.6(2/55)	18.4(7/38)	0.029*
Cage Subsidence (%)	1.8(1/55)	28.9(11/38)	0.001*

ADH, anterior disc height; MDH, middle disc height; PDH, posterior disc height; FH, foraminal height; SLA, segmental lordosis angle. * P < 0.05, difference was significant

## Discussion

Previous studies have shown that OLIF for degenerative lumbar disease treatment is a viable alternative to P/TLIF with significant improvement of clinical results [14]. Patients in this study exhibited significant improvement in VAS, ODI, and JOA scores at the last follow-up, suggesting that OLIF successfully alleviated the clinical symptoms. Indirect decompression in OLIF was achieved through the increased DH. The A/M/P DH was increased by 4.6, 3.0, and 1.6 mm postoperatively in this study. Similar to the findings of Shimizu et al., a mean 2.6 mm and 1.5 mm increase in M/PDH was found with the cross-sectional area increasing from 136.4mm^2^ to 194.1mm^2^ [22].

The SLA significantly increased with the insertion of the interbody cage. Postoperative SLA is significantly associated with ASD [2–4] and a SLA increased more than 5° could effectively reduce the risk of ASD (OR: 0.454, P < 0.01) [1]. Although various factors associated with the restoration of SLA were reported in previous study, the present study demonstrated through multifactor analysis that restoration of the SLA in OLIF was dependent on cage position, preoperative SLA and cage subsidence.

Better restoration of the SLA could be achieved by placing the cage in the anterior position and Park et al. recommended the anterior 1/3 of the intervertebral space as the ideal position for placement of the cage in LLIF due to better restoration of the SLA with successful indirect decompression [23]. Qiao et al. also reported cages located at the anterior 1/3 with better SLA improvement than those at the posterior 2/3 (2.8° versus 0.8°) in LLIF [24]. However, an anteriorly placed cage (0–20%) with the highest risk of endplate injury in OLIF and a cage located at 20–40% anteriorly were recommended. In this study, the CPR was used to assess the cage position, which was evaluated based on the ratio in lateral X-ray [20]. A previous study showed that the CPR was strongly positively correlate with increased SLA(r = 0.597, *P* < 0.01) in P/TLIF [15]. In this study, the CPR was significantly positively correlated with increased SLA (r = 0.526, *P* < 0.01) and cages placed at CPR ≥ 50% could achieve better intraoperative SLA increases with a lower risk of intraoperative endplate jury (5.9 ± 3.9° versus 4.2 ± 3.2°, *P* < 0.05, 3.6% versus 18.4%, *P* < 0.05, respectively).

Restoration of the SLA was negatively correlated with the preoperative SLA (r=-0.575, *p* < 0.01) in this study which was similar to the results of Liu et al. who found that SLA restoration was dependent on the preoperative SLA after TLIF [25]. We believed the change in SLA from the less lordotic or kyphotic segment to the neutral or lordotic segment was higher than that with the lordotic segment. For lordotic segments, it is important to maintain the original SLA.

A significant loss of SLA was observed at the last follow-up in this study. Cage subsidence was the main factor affecting the SLA and DH loss reported in previous study [17, 26] and in the present study, the loss of SLA was significantly larger in the subsidence segment. Previous research has reported multiple risk factors associated with cage subsidence such as BMD, inappropriate cage height and cage position [21, 27–31]. The results of this study were similar to those of previous studies showing that L1 CT HU < 110, cage height > preoperative MDH, and CPR < 50% were significantly associated with cage subsidence.

Patients with T<-2.5 showed a higher risk of developing cage subsidence [27], however, the T-score provided by dual-energy X-ray absorptiometry may underestimate osteoporosis in patients with lumbar degenerative disease. L1 CT HU ≤ 110 for defining osteoporosis with high specificity and sensitivity [18] and Wu et al. reported that patients with lower CT HU were prone to intraoperative endplate injury and delayed cage subsidence but this did not affect the clinical outcomes [27]. Pisano et al. reported that the subsidence segment showed significantly smaller L1 CT HU than the nonsubsidence segment in TLIF (137.71 vs. 167.8 HUs, *P* = 0.002) [32]. And selecting a cage height according to preoperative DH was recommended for preventing risk of subsidence. Kotheeranurak et al. found that selection of a large cage height (12 mm) was reported as risk factor for cage subsidence (OR: 9.588, *P* < 0.01) in OLIF [30]. We choose the cage height based on the preoperative DH at surgical and adjacent segment because of previously study found cage height > 1.3 mm above the height of the suprajacent level is a risk factor for cage subsidence[30]. In this study, cage height > preoperative MDH was found in 53 (57.0%) level with a higher risk of cage subsidence (2.5% versus 20.8%, *P* = 0.011).

Additionally, Singhatanadgige et al. reported that posterior cage position was a risk factor of cage subsidence (OR = 4.2; p = 0.005) [28] and in the present study, cages placed in the anterior position (CPR ≥ 50%) significantly reduced the incidence of cage subsidence (1.8% versus 28.9%, P < 0.05). We thought that the anterior edge of the cage in the anterior position could span the anterior apophyseal ring which had a higher density than the middle portion of the endplate. However, the anterior edge of the cage in the posterior position was located at the middle position which was the weakest position with the highest migration rate [33], causing a significantly higher risk of intraoperative endplate violation and reduction of SLA. Even in the intraoperative endplate violation segment, the cage placed in the anterior position showed significantly lower SLA decreased at follow-up (1.0° vs. 4.3°).

Combining our findings with those of previous studies, we identified the cage position as the most critical determinant of SLA restoration in OLIF. A cage placed in the anterior position (CPR ≥ 50%) could extend the anterior side with solid support, and it did not affect the indirect decompression outcomes. Mahatthanatrakul et al. reported that the position of the cage does not affect the indirect decompression while cage placed in a more posterior position correlated with increase in FSCA in OLIF through a median 12 months (interquartile range, 8.25; range, 6 to 41 months) follow-up [34]. However, Qiao and Park’s study showed there were no significant differences in the FH, foraminal area, and the cross-sectional area of the thecal sac increased with respect to the cage position [23, 24]. This study found that the increase in FH was significantly higher in the posterior group at post-operatively, butthere was no significant difference between the two groups at the last follow-up because the higher risk of cage subsidence in the posterior group may undermine the correction.

There were several limitations in this study that should be considered when interpreting the results. The width and angle of the cage may also affect the restoration of the SLA and the incidence of cage subsidence. However, all the fusion devices included in this study were 22 mm in width and 6°, which requires further evaluation of the impact of the width and angle of the cage on the restoration of lumbar sagittal alignment [35, 36]. Additionally, a prospective study with a larger sample size and a long follow-up are needed to confirm the reliability of the new scoring system.

## Conclusion

Restoration of the SLA in OLIF was dependent on preoperative SLA, cage position and cage subsidence. Placement of the cage in the anterior portion of the intervertebral space (CPR ≥ 50%) was the key determinant of SLA restoration due to a large SLA increase and reduced the risk of cage subsidence.

## Data Availability

The datasets will be available from the corresponding author if required.

## Abbreviations

OLIF: Oblique lumbar interbody fusion
PLIF: Posterior lumbar interbody fusion
TLIF: Transforaminal lumbar interbody fusion
LLIF: Lateral lumbar interbody fusion
ALIF: Anterior lumbar interbody fusion
SLA: Segment lordosis angle
DH: Disc heights
FH: Foraminal height
VAS: Visual analog scale
ODI: Oswestry Disability Index
BMD: Bone mineral density
CPR: Cage center point ratio (CPR), CT HU:Computed tomography Hounsfield Unit
